# History of an Insidious Case of Metastatic Insulinoma

**DOI:** 10.3390/jcm14124028

**Published:** 2025-06-06

**Authors:** Katarzyna Antosz-Popiołek, Joanna Koga-Batko, Wojciech Suchecki, Małgorzata Stopa, Katarzyna Zawadzka, Łukasz Hajac, Marek Bolanowski, Aleksandra Jawiarczyk-Przybyłowska

**Affiliations:** 1 Student Scientific Group of Endocrinology, Wroclaw Medical University, Wybrzeże Pasteura 4, 50-367 Wroclaw, Poland; antosz0715@gmail.com (K.A.-P.); joanna.batko99@gmail.com (J.K.-B.); wsuchecki881@gmail.com (W.S.); stopa98@gmail.com (M.S.); 2 Department and Clinic of Endocrinology and Internal Medicine, Wroclaw Medical University, Wybrzeże Pasteura 4, 50-367 Wroclaw, Poland; katarzyna.zawadzka@umw.edu.pl (K.Z.); aleksandra.jawiarczyk-przybylowska@umw.edu.pl (A.J.-P.); 3 Neuroendocrine Cancer Unit, Lower Silesian Oncology, Pulmonology and Hematology Center, 50-367 Wroclaw, Poland; hajac.l@dco.com.pl

**Keywords:** insulinoma, metastases in insulinoma, long-term complicated insulinoma, rare tumor

## Abstract

In this article, we present a case of a 49-year-old woman presenting with a recurrent metastatic neuroendocrine tumor. **Background**: Insulinomas are neuroendocrine tumors derived from beta cells of the pancreas that secrete insulin. Usually, they are benign tumors; however, metastatic insulinomas are an extremely rare malignant form of these tumors, carrying a significantly worse prognosis. **Case Presentation**: A 49-year-old woman, a patient in the University Hospital in Wroclaw in the Department of Endocrinology, Diabetes and Isotope Therapy, first presented with abdominal pain in 2009, when ultrasound and further examination led to the diagnosis of a tumor in the pancreas (a solid pseudopapillary tumor of the pancreas—meta NET G2), and the patient underwent distal pancreatectomy with splenectomy. For ten years, she was under observation, and her symptoms, such as abdominal pain, nausea, weight loss, and general weakness, reappeared in 2019. Then, magnetic resonance imaging (MRI) showed a lesion in the liver, and further histopathology revealed neuroendocrine tumor (NET) metastasis to the liver. In 2022, the patient presented with loss of consciousness and convulsion, loss of weight, and hypoglycemia after meals. In April 2022, the daily glycemic profile was recorded and a 72 h fasting test was performed; however, their results excluded insulinoma. Positron emission tomography–computed tomography (PET-CT) with 18F-fluorodeoxyglucose (18F-FDG) and PET with gallium-68-DOTA-(Tyr3)-octreotate (68Ga-DOTA-TATE) showed a metastatic proliferative process in the liver. Persistent hypoglycemia led to another hospitalization in May 2022, and repeated tests allowed for the diagnosis of insulinoma. Treatment with somatostatin analogs and diazoxide was started. A CT scan in November 2022 and a PET scan in January 2023 showed new metastases to the liver, bones, and cervical lymph nodes, and it was decided to intensify the treatment. In May 2023, the patient was qualified for Lutathera treatment for insulinoma at the University Clinical Hospital in Poznań. In June 2023, another disturbing symptom was reported by the patient, a painful lump in the breast. During diagnostics, metastases with high proliferation markers were found in both breasts. Two months later, in August 2023, the patient received another dose of Lutathera. In October 2023, significant progression of liver lesions, metastases to bones of the spine, ribs, and pelvis, and periaortic and pelvic lymphadenopathy were found as well as elevated values of neuron-specific enolase and calcitonin. The patient was also referred to the Palliative Medicine Home Hospice. In consultation with the Lower Silesian Cancer Center, the decision was made to forgo further treatment with PRRT and initiate systemic chemotherapy. Despite the chosen treatment, the patient died on 27/DEC/2023. **Conclusions**: This case report can serve clinicians, as it presents a case of an extremely rare and insidious tumor, metastatic insulinoma.

## 1. Introduction

Insulinoma represents the most common type of pancreatic neuroendocrine tumor. However, it is still a very rare tumor, with an occurrence ratio ranging between one and four people per million annually; nonetheless, according to some autopsy studies, these numbers are underestimated [1,2]. A vast majority of insulinomas occur in sporadic settings, and only about 5 to 10% of cases are associated with genetic syndromes [3]. Sporadic tumors typically present as unifocal, benign, and well-encapsulated lesions measuring less than 2 cm in their major axis [4,5].

Clinical symptoms are variable but usually include hypoglycemia as the most prominent one. Some tumors, especially those of a small size, might be clinically silent, prolonging the time before diagnosis. The “Whipple triad”—signs of hypoglycemia, plasma glucose levels below 3 mmol/L, and resolution of symptoms after glucose administration—is considered pathognomonic for insulinoma [3,6,7]. Diagnosis is made upon laboratory findings based on glucose and insulin interaction and imaging methods. Typically, a 72 h long fasting test is performed, during which levels of glucose, insulin, C-peptide, and, if possible, proinsulin and beta-hydroxybutyrate are assessed. A combination of results—glycemia <2.5 mmol/L, insulin levels >20.8 pmol/L, and C-peptide levels >0.2 nmol/L—is highly indicative of the presence of an insulinoma [3,6]. To localize the tumor precisely, invasive methods are helpful, such as endoscopic ultrasonography and somatostatin receptor scintigraphy. Over 99% of insulinomas are located within the pancreas, evenly distributed across the head, body, and neck [8]. The gold standard and only curative treatment method for non-metastatic tumors is surgery.

However, only about 10% of insulinomas present with malignant behavior, making these tumors exceptionally rare [9]. Malignancy is defined by locoregional invasion or metastases. The limited number of cases and scarce data fail to provide adequate guidelines regarding clinical presentation, preferred treatment modalities, and outcomes for malignant insulinoma. Malignant tumors generally have a poorer prognosis with a median survival period of approximately 2 years [9,10]. Metastases are mostly found in the lymph nodes and liver, rarely involving the bones, brain, peritoneal tissue, and lung [6,11]. A larger tumor size and increased serum β-cell polypeptide might be indicators of malignancy, but there are no definite criteria for predicting patients who might develop metastatic insulinoma [12,13]. Operative strategies are not likely to be curative in metastatic tumors. Management of non-resectable liver metastases consists of locoablative techniques, such as transcatheter embolization, transcatheter arterial chemoembolization, radiofrequency thermal ablation, and ethanol ablation [14]. To alleviate the symptoms of hypoglycemia, diazoxide and somatostatin analogs are used [15]. Other treatment options include mammalian target of rapamycin (mTOR) inhibitors, tyrosine kinase inhibitors, and the recently studied peptide receptor radionuclide therapy [16].

The case of a 49-year-old woman with a recurrent metastatic neuroendocrine tumor is presented below.

## 2. Case Description

The patient’s history started with disturbing symptoms in 2009. As a first sign, abdominal pain was reported, and a pancreatic tumor was described during an ultrasound (US) examination. Along with further examinations, the patient was qualified for surgery, specifically distal pancreatectomy with splenectomy. The histopathology test indicated a solid pseudopapillary tumor of the pancreas—meta NET G2 (Table 1). Genetic tests for mutations (BRCA1, BRCA2, deletion in CHECK2) were also performed, which were negative. She spent 7 years under the care of a hospital abroad. For the following three years, the patient remained under no control.

Later, in July 2019, she again complained of abdominal pain and fatigue, which led to an indication in the MRI and intense contrast enhancement with dimensions of 47 × 37 mm. The patient underwent segmentectomy (S5-S4b) with cholecystectomy (16/SEP/2019) with the histopathological recognition of neuroendocrine tumor (NET) metastasis to the liver characterized as NET G2, Ki67—5%, with positive (+) chromogranin A, synaptophysin, cytokeratin, and CD56, and negative (−) TTF-1 and CDX-2.

Due to her change of residence and moving to Poland, she was taken care of by the Lower Silesian Pulmonology and Hematology Oncology Center, where abdominal and thorax computed tomography scans with contrast and abdominal MRI demonstrated no sign of metastasis (from 2020 to 2021). In laboratory tests, markers such as chromogranin A and NSE were also within the norm. Later, in February 2022, somatostatin receptor scintigraphy was performed (SRS)—with the result of an area of focally increased tracer accumulation in the caecum with wall thickening of the large intestine. Due to these results, a colonoscopy was performed; however, it did not indicate any form of pathology. Moreover, one month earlier, in January 2022, a short episode of loss of consciousness with convulsion was reported. She experienced the initial symptoms of dizziness and blurred vision. The glycemia outcome was 57 mg%. She was hospitalized at the Neurology Department due to a seizure caused by hypoglycemia. The patient was ordered to independently monitor glucose levels in her blood. The results of these measurements were from 45 mg% to 300 mg%, with the lowest levels after meals. Although she tried to eat every two hours, she lost 7 kg and suffered from intermittent nausea and, as before, abdominal pain. Additionally, the patient slept soundly at night, eating dinner around 8 p.m.

According to the recurrent states of hypoglycemia, she was hospitalized in the Department of Endocrinology, Diabetes and Isotope Therapy in Wrocław in April 2022. The daily glycemic profile indicated low glycemia values up to 37 mg%. A 72 h fasting test was performed, and in the time of the 72 h fast, no states of hypoglycemia were reported, excluding insulinoma from further diagnosis. The concentrations of chromogranin A, gastrin, and NSE were also within normal values. Regarding the abnormal results, calcitonin was significantly above the upper limit of normal (Table 2). Ultrasound of the thyroid gland was normal, without any tumor. The results of blood tests indicated the correct pituitary function in all tropic axes, including the adrenocorticotropic and somatotropic axes. Additionally, the concentration of HbA1C was elevated (7.1%). A prolonged oral glucose tolerance test was carried out, in which the patient, after a glucose load, showed an extremely high glucose level in her blood after 2 h (375 mg/dL). Diabetes type 3 due to partial pancreatectomy was diagnosed (Table 3). She was discharged from the hospital with the recommendation to monitor her glucose levels and, depending on the daily glycemia, with an SGLT-2 inhibitor to consider.

**Figure 1:**
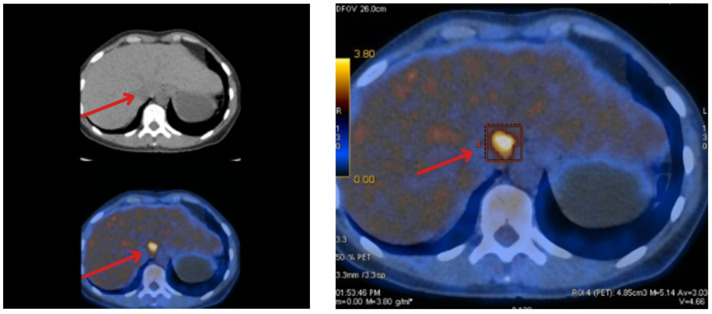
PET-CT was performed in April 2022. The picture shows the focal point of increased glucose metabolism in segment I of the liver, indicated by arrow (SUV max = 4.6, in delayed SUV max = 5.1). (**Left**) shows a comparison of CT and PET images. (**Right**) The image shows the enhancement in PET imaging.

Due to the visualization of individual liver calcifications during US, PET-CT with 18F-FDG was also performed (Figure 1; 14/APR/2022) and showed increased metabolism of FDG in segment I of the patient’s liver, which related to the metastatic proliferative process in this area. PET with 68Ga-DOTA-TATE (21/APR/2022) showed a focus on moderately elevated expression of somatostatin receptors in the projection of the caudate lobe of the liver adjacent to the inferior vena cava and an area of the moderately elevated tracer accumulation in the projection of the ascending part of the duodenum, the dimensions of which were about 9 mm, of a probable functional nature.

Due to persistent hypoglycemia in individual measurements and the very suggestive clinical course of the patient, it was decided to conduct further tests. She was submitted to the Department of Endocrinology, Diabetes and Isotope Therapy in Wrocław on 12/MAY/2022 to repeat the diagnostic process toward insulinoma. Already on the first day of hospitalization, glycemia dropped to 33 mg/dL, which was associated with higher insulin and C-peptide levels. The results finally confirmed the diagnosis by fulfilling the criteria for insulinoma. The criteria, already mentioned in the introduction, are described as a combination of clinical symptoms of hypoglycemia along with glycemia < 2.5 mmol/L (45 mg/dL), insulin levels > 20.8 pmol/L, and C-peptide levels > 0.2 nmol/L. The laboratory test results are presented in Table 4.

On 16/MAY/2022, a test with short-acting octreotide was performed to assess the possibility of using somatostatin analogs (Table 5). Suppression of insulin secretion was observed; therefore, the test was considered diagnostic, and treatment was started with a dose of 20 mg. During the first day, after drug administration, a decrease in blood glucose levels during the night, especially at about 3.00 a.m., was noted; therefore, it was decided to include diazoxide (1 tablet in the evening) in the treatment.

An abdominal MRI was also performed, and it showed a tumor measuring 26 × 24 × 29 mm located next to the inferior vena cava in segment I of the liver, showing diffusion restriction. The tumor was characterized by a heterogeneous contrast enhancement in its central part. After administration of the contrast agent, the tumor rim became stronger first, and after that the center. Retrospectively comparing this to the previous MRI, the tumor had enlarged (former dimensions from 23.08.21: 20 × 15 × 20 mm).

Due to decreased levels of fT4 (8.46 pmol/L, normal range: 9.01–19.05) and IGF-1 (87.1 ng/mL (n. 94–252), an MRI of the pituitary gland was performed two months later, but no abnormalities in the pituitary–hypothalamic area were found. However, another gradual weight loss had already been observed.

To determine further management, gastroscopy and EUS were directed. Gastroscopy indicated the erythematous mucosa. EUS with a biopsy of a heterogeneous area in the left lobe of the liver from 01/JUN/2022 revealed results showing the presence of malignant neoplasm cells. The morphology and immunohistochemistry type corresponded to a neuroendocrine tumor with higher malignancy, G2/G3 (CK19 (+), PanCK (+), synaptophysin (+), Ki67 up to 20%). With these results, the patient was admitted to the Department of Oncologic Surgery at the Lower Silesian Cancer Center to receive further therapy. On 25/AUG/2022, a median upper relaparotomy with alcoholization of the liver tumor was performed. The patient was discharged home in a good general condition.

About a month later, the patient was referred to the Department of Endocrinology, Diabetes and Isotope Therapy in Wroclaw for follow-up. A glycemic profile with a single drop in glycemia to 48 mg/dL and abnormal fasting glucose levels (128 mg/dL to 207 mg/dL) was presented. Further treatment with a somatostatin analog (octreotide LAR 30 mg once every 28 days) was recommended following the hospitalization without diazoxide.

Two months later, the patient underwent a follow-up CT scan of the chest and abdomen. Unfortunately, the examination showed new foci of liver metastases: in S1 (2.6 × 2.0 cm in diameter), S2 (0.8 cm and 0.7 cm in diameter), S3 (0.7 cm in diameter), and S6 (1.2 cm in diameter).

In January of the following year, the patient went for a follow-up PET scan at the Świętokrzyskie Oncology Center (ŚCO). Compared to the previous study from April 2022, a significantly increased expression of somatostatin receptors was shown in the cast of liver S1 (SUV max. 9.9). In addition, a new concentration of the tracer was visualized—mainly in the left lobe of the liver (SUV max. 5.5). There was a thickening of the tracer in the head of the pancreas (SUV max. 4), single foci in the bones (SUV max. 4.9), and elevated receptor expression in two cervical lymph nodes (lower left, group IV, SUV max. 1.5). Based on these results, a decision was made to intensify the treatment with a somatostatin analog: lanreotide 120 mg every 2 weeks (from February 2023).

**Figure 2:**
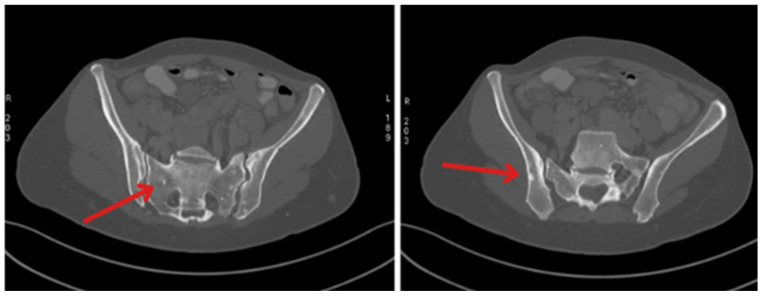
CT recorded on 23/MAY/2023 showing bones with airy areas of discrete sclerotization up to 1.2 cm in size and numerous punctate foci of sclerotization (indicated by arrows).

In May 2023, the patient was qualified for Lutathera treatment for insulinoma at the University Clinical Hospital in Poznań. On 12/MAY/2023, [17Lu]-DOTATATE with an activity of 200 mCi was administered with nephroprotection. The patient was discharged in good condition (body weight—56 kg; height—160 cm; BMI—21.87 kg/m^2^) and advised to visit again in three months for another dose of Lutathera. Two weeks after the first dose of later Lutathera, a comparative CT scan was performed. Liver enlargement was shown. The right lobe of the liver in the CC dimension reached 16.5cm, while the left lobe reached the left subdiaphragmatic region and modeled the adjacent organs. The left lobe was nodularly altered—multiple and confluent areas of metastasis reaching up to 6.4 cm × 5 cm (TR). The right lobe also visualized multiple metastatic lesions up to 3.7 cm × 3.2 cm in size. In addition, thickened visceral adipose tissue of the left epigastrium (up to about 1.3 cm thick) with an infiltrative/edematous nature was seen. The left adrenal gland was thickened to 1.2 cm. Numerous areas of discrete sclerosis (up to 1.2 cm in size) were visualized in the bones of the scope and were defined as suspected pathological remodeling for observation (Figure 2). Two slightly larger lymph nodes (probably reactive), up to 1.3 cm in size on the short axis, were also seen in the left supraclavicular region.

On 12/JUN/2023, the patient was admitted to the Department of Endocrinology, Diabetology and Isotope Therapy in Wroclaw. Due to fluctuating glycemic levels, follow-up laboratory tests were planned. Biochemical tests showed elevated glycated hemoglobin (HbA1c 7.7%), fasting glucose (141 mg/dL), and LDH (285 U/L), and decreased hemoglobin (10.9 g/dL). The results of the test markers associated with NETs are presented in Table 6. Given the high daytime glycemic values (especially postprandial), the decision was made to set up a flash glucose monitoring system (FGM) and use fast-acting postprandial insulin (Insulin Aspart 2 units for a meal).

Additionally, due to a painful lump in the right breast, an ultrasound was carried out, which imaged a focal solid lesion in the right mammary gland—BIRADS 4A and cysts in both breasts (13/JUN/2023). Mammography was performed (29/JUN/2023), with the results of the left breast showing multiple cysts up to 7 mm in size (BIRADS 2). No pathology was visualized in the left axillary area. However, in the right breast at 9 o’clock (5 cm from the nipple), a hypoechoic focus of 10 mm × 5 mm in size and 6 mm in depth was found. A pathological lymph node of 17 mm in size was seen in the right axillary area. The lesion was classified as BIRADS 4B, and a biopsy of the right breast lump was performed with the result of invasive ductal carcinoma G2. Subsequently, a left breast biopsy was also performed (14/AUG/2023). Histopathological examination revealed a malignant, invasive neoplasm with neuroendocrine differentiation (infiltrating duct carcinoma subtype). The grade of differentiation was G3. No estrogen, progesterone, or HER2 receptors were found. After comparing the histopathology of the liver and breast metastasis, it was confirmed that these were the same cells from the patient’s primary tumor with further dedifferentiation. Additionally, the detected metastases were characterized by increasingly higher markers of tumor proliferative activity. Markers are presented in Table 7.

On 21/AUG/2023, the patient went to the University Clinical Hospital in Poznań to receive the following dose of Lutathera. The patient weighed 49 kg at that time, was 160 cm tall, and had a BMI of 19.14 kg/m^2^. At the same time, preparations were started for systemic treatment of breast cancer. For this purpose, echocardiography was performed with no cardiac contraindications to oncological treatment.

**Figure 3:**
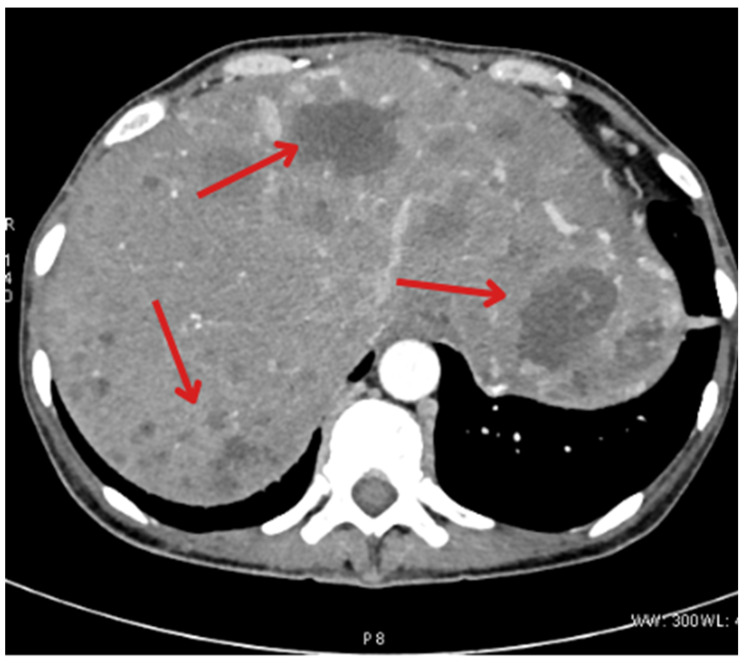
An October abdominal CT scan revealed further progression of metastatic lesions to the liver. The liver is enlarged with very numerous, uncountable metastatic foci with features of disintegration (indicated by arrows).

**Figure 4:**
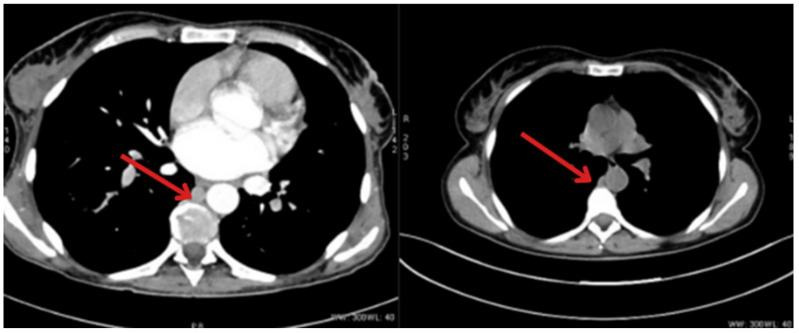
The same study found periaortic lymphadenopathy, indicated by arrows.

During the follow-up hospitalization at the leading Department of Endocrinology in Wroclaw from 31/OCT/2023 to 4/NOV/2023, the patient complained of severe pain, mainly in both breasts, and analgesic therapy was administered (tramadol and dexketoprofen). Additionally, a contrast-enhanced CT scan of the abdomen was performed (Figure 3). Significant progression of liver lesions was found, compared to the previous examination—the liver was significantly enlarged with uncountable metastatic lesions. The left adrenal gland was slightly thickened. Highly abundant metastases to the bones of the spine, ribs, and pelvis were visible (the largest osteosclerotic focus in the L1 vertebral body was 1 cm × 0.7 cm). Periaortic and pelvic lymphadenopathy (Figure 4) and peritoneal dissemination were also found. During the hospitalization, elevated values of neuron-specific enolase (388.4 μg/L) and calcitonin (2000 pg/mL) were found, which were associated with massive dissemination of cancer. Due to anemia (8.8 g/dL) and its successive decrease, a decision was made to transfuse 2 units of red blood cell concentrate.

**Figure 5:**
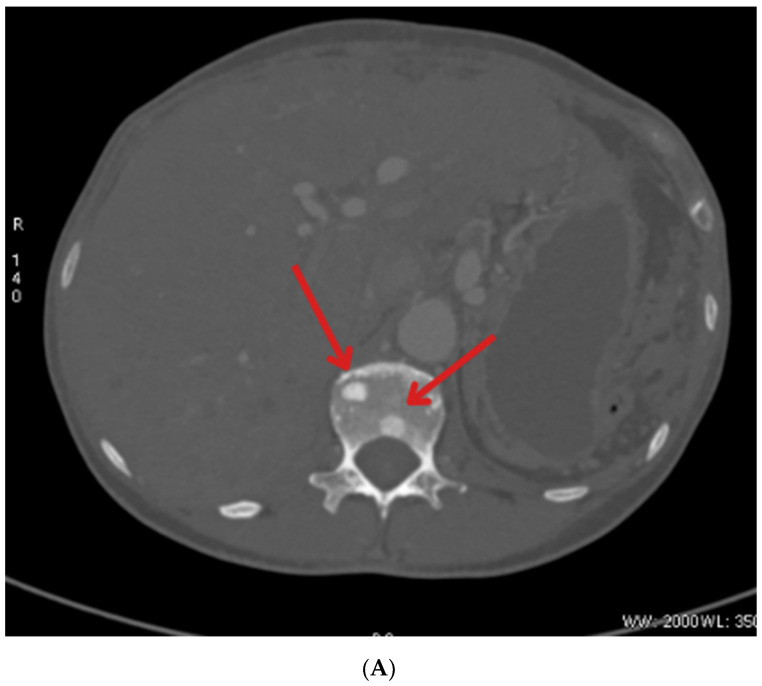

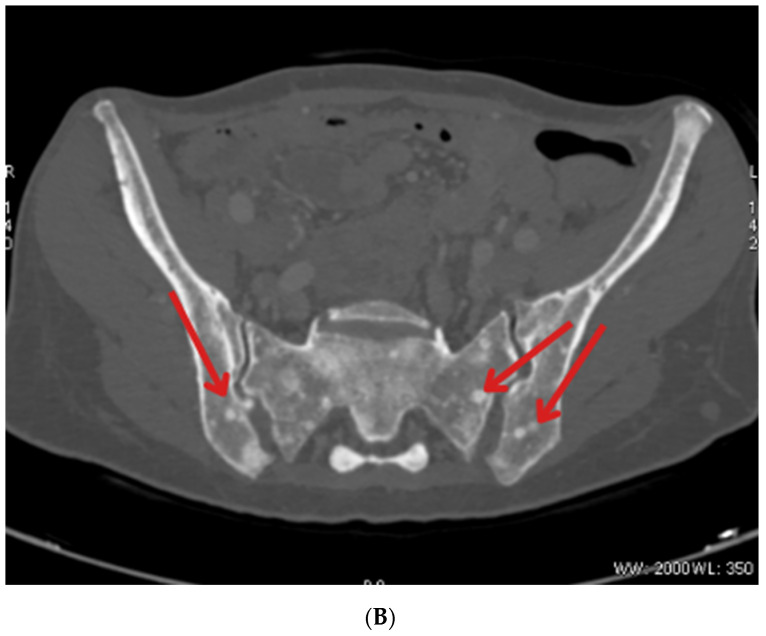
CT also visualized very abundant metastases in the bones of the spine (**A**), ribs, and pelvis (**B**)—the largest osteosclerotic focus in the L1 vertebral body measuring 1 cm × 0.7 cm, (metastases indicated by arrows).

In connection with the above, the patient was referred to the Palliative Medicine Home Hospice for control of pain treatment. Due to the progression of the lesions found on the current CT scan (Figure 5), in consultation with the Lower Silesian Cancer Center, the decision was made to forgo further treatment with PRRT and initiate systemic chemotherapy. The patient was given one course of temozolomide metronomically (75 mg/m^2^ daily week on/off). Unfortunately, further progression of very advanced disease and deterioration of patient status led to death on 27/DEC/2023.

## 3. Timeline

**Figure 6:**
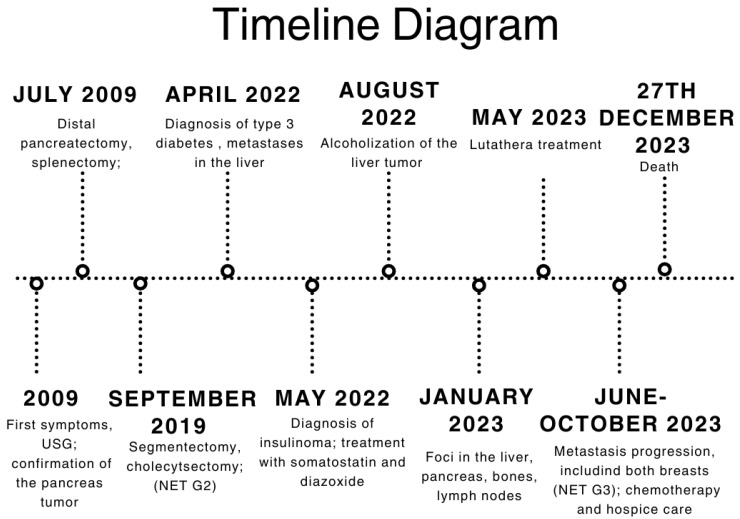
A timeline diagram of the most crucial turning points of diagnosis and treatment. The diagram was created as a simplified visualization of the table above, where other information can be found.

To improve access to specific stages of diagnosis and treatment, and to make the findings of individual key elements of the patient’s history easier to understand, a table and timeline have been prepared (Table 8 and Figure 6).

## 4. Discussion

According to the timeline above, a discussion about this case is required. From the beginning of 2009, when the first diagnosis was made, the patient struggled with most complications.

Looking at the first results, a solid pseudopapillary tumor of the pancreas, known as a Frantz tumor, was diagnosed. This variation is a rare type of pancreatic tumor, strongly predominant in young women around the second and third decades of life, with a clinical history of very few men cases. From this very first step of the patient’s history, it is a rather infrequent case. Moreover, solid and papillary tumors of the pancreas (SPTs) are thought to account for 1–2% of exocrine pancreatic tumors, and are also mostly described as non-malignant. As a treatment, complete resection is associated with long-term survival, although different types of procedures have been performed and recommended over the years [17,18,19]. In this case, a distal pancreatectomy with splenectomy was performed. At this point, it is crucial to point out the gold-standard treatment for localized insulinoma, which is complete surgical resection. The method of resection, from enucleation and distal pancreatectomy up to the Whipple procedure, is determined based on the surgeon’s assessment, taking into account the size, location, and aggressiveness [1].

Following the patient’s history over the next 10 years, the patient did not experience any significant ailments. A lesion in the liver was found with the occurrence of new symptoms [12,20,21]. The patient remained symptom-free for over a decade until a liver lesion was discovered alongside the emergence of new symptoms. By January 2022, the patient exhibited typical insulinoma symptoms, including neuroglycopenic and autonomic symptoms affecting 60–100% of patients [20,21].

As stated in the case description, the fasting test performed during the hospital stay was negative. The fasting test has some limitations. In people with iatrogenic hypoglycemia or calcium metabolism disorders, the test results may be abnormal. In contrast, the sensitivity of the test is within 90%. Therefore, negative test results do not entitle us to confidently reject the diagnosis of insulinoma [22].

During the patient’s regular follow-up appointments at the Department of Endocrinology, Diabetes and Isotope Therapy and the Lower Silesian Pulmonology and Hematology Oncology Center, the result of NSE was more than three times the normal values (75.85 μg/L). Gamma-enolase is one of the isoenzymes of enolase, derived from mature neuronal cells. Its concentration is particularly useful in long-term monitoring of the course of insulinoma. Its level varies with the size of the tumor mass, making it a good marker of treatment effects [23]. Furthermore, due to its high specificity, it can be used as a stand-alone marker in the surveillance of insulinoma [24].

When the diagnosis of insulinoma was made, the patient was at a point where metastases were present in the liver. This situation required the initiation of pharmacological treatment. The most important aspect was to prevent states of severe hypoglycemia. This can be achieved with the use of somatostatin analogs (SSAs) like octreotide, lanreotide, or diazoxide—an antihyperglycemic drug that acts through opening potassium channels on β-pancreatic islet cells, leading to decreased insulin secretion, streptozocin, verapamil, or phenytoin [15]. The treatment prescribed to the described patient was an SSA and diazoxide, which represent the first-line therapy used to control both clinical symptoms and proliferative disease, especially in the case of patients with large drops in glycemia, as both substances act synergistically [25].

It is estimated that about 70% of insulinoma tumors present receptors for somatostatin, which inhibit pancreatic extra- and endocrine function, thus contributing to a reduction in the severity of clinical symptoms [26]. Improvements in the course of the disease after the use of SST analogs have been observed in 30% to 85% of patients, and reductions in the concentrations of tumor markers have been observed in up to one in two patients [27]. As a result, the patient was taking Sandostatin LAR (octreotide).

Due to the progressive disease, the patient was also enrolled in a peptide receptor radionuclide therapy (PRRT) program using Lutathera (Lu177-dotatate)—200mCi of the preparation at four-month intervals. PRRT is approved for all types of NENs expressing somatostatin receptors, showing antitumor efficacy in patients with advanced gastroenteropancreatic neuroendocrine neoplasms (GEP NENs) [25]. PRRT, which is usually considered a safe treatment, has shown greater efficacy in managing metastatic neuroendocrine tumors compared to octreotide alone [28]. It also causes effective resolution of hypoglycemia, thereby significantly improving patients’ quality of life [29,30,31,32]. Despite PET-CT with 18F-FDG (+), the NETPET Score was taken into account, which is a grading system where the results from the combined reading of SRI-PET and FDG-PET are reported as a single parameter. According to the NETPET Score, there was a chance to achieve effects from radioligand treatment, and therefore it was the procedure of choice [33,34].

Attempts were also made to treat the cause—to reduce the volume of the tumor by removing its hepatic metastases. For this purpose, the method of choice was alcohol ablation of the tumor. Alcoholization of insulinoma and liver metastases, in patients with an increased risk of complications of classical surgery, allows us to perform a less burdensome tumor alcoholization procedure for the benefit of the patient [35].

The last line of treatment that was used in the described patient, and which has not been discussed yet, was chemotherapy. Chemotherapy remains the treatment of choice for advanced progressive functional NENs or in high-grade NET G3, as in the discussed case [25]. Many possible drug combinations are available; in this case, the Lower Silesian Center for Oncology, Pulmonology, and Hematology decided to use temozolomide administered systemically while disqualifying the patient from further PRRT therapy.

Concluding the discussion of the therapeutic interventions undertaken, it is worth mentioning one more line of treatment, which, although considered, was not implemented. Everolimus is an oral m-TOR inhibitor that was among the targeted agents considered [25]. Unfortunately, due to the rapid spread of the disease, increasingly high K67 values, and the G3 disease stage, this drug was never used, and chemotherapy was attempted.

Referring to the patient’s focal solid lesion in the right mammary gland (described as BIRADS 4A), histopathological examination revealed a malignant, invasive tumor with neuroendocrine differentiation (infiltrating duct carcinoma subtype). At this point, insulinoma-associated protein 1 (INSM1) expression is often found in pancreatic neuroendocrine tumors. In a study by Kim et al. [36], nuclear staining of INSM1 was demonstrated in all cases of 55 PNET patients. In addition, it has been shown that INSM1, by regulating the activity of C-MYC and p-ERK pathways, can promote breast cancer development [37], and a large clinical trial recommends INSM1 in cases of IBC with neuroendocrine differentiation [38].

It is also worth highlighting the strong tendency to metastasize in this case. As the disease progressed with time, the patient was subjected to regular follow-up imaging examinations. The cancer was spreading more and more. In addition to the liver metastasis, after 4 years, PET imaging showed foci of metastasis in the bones. There are isolated cases reported in the literature regarding insulinoma metastasis to bone. However, they are very rare and involve only the most aggressive courses of the disease [12,21,39]. Tracer thickening has also been demonstrated in the cervical lymph nodes. Simultaneous metastasis to lymph nodes and the liver occurs in only 5–12% of reported cases of insulinoma [40]. In order to better illustrate and present the remaining described cases of patients suffering from different forms of insulinoma, a table has been prepared. Table 9 describes patients with and without metastases, emphasizing the differences in treatment and prognosis.

Last but not least, the perspective of subsequent histopathological verifications over time shows the process of dedifferentiation of the tumor (initially G2 Ki67 of 5%, then 20% in the liver, then already dedifferentiated cancer in the breast with Ki67 of 30% and 50% for left and right breasts, respectively) and its role is crucial in therapeutic decisions. The confirmation of dedifferentiation of the disease can significantly affect the decision on the type of systemic treatment—RLT vs. targeted therapy vs. chemotherapy.

## 5. Conclusions

It must be emphasized that the general clinical picture of this patient indicates a very aggressive course of the tumor. The described hepatic, bone, and breast metastases were significantly progressive, as only five months later in the clinical history, new liver metastases and potential foci of metastasis to the fatty tissue of the left epigastrium and adrenal gland were found on a CT scan.

This is an example of very rapid growth of the tumor, which is not usually associated with such a tendency. Awareness of the aggressive form of insulinoma is essential for proper treatment and control.

For other clinicians, this case may serve as an example of an exceptionally insidious insulinoma.

## Figures and Tables

**Table 1:** The pathological report from the surgery in 2009: (+)—positive; (−)—negative; (+/−)—slightly positive. Results were obtained from a copy of a discharge summary from a hospital in Kiev.

Types of Test	Outcome
Vimentin Ab2	+/−
Cytokeratin Clone AE1/AE3	−
Cytokeratin 7	−
Chromogranin A	+
Neuron-Specific Enolase (NSE)	+
Synaptophysin	+

**Table 2:** Results of significant (blood) neuroendocrine markers from the first hospitalization in the Endocrinology Department.

Marker	Result	Normal Range
Chromatogranin A	43.2 µg/dL	0–100 µg/dL
Gastrin	69.7 pg/mL	13–115 pg/mL
NSE	13.3 µg/dL	0–18.3 µg/dL
Calcitonin	**104 pg/mL**	0–11.5 pg/mL

**Table 3:** Prolonged oral glucose tolerance test—the dose of glucose taken is 75 g. The norms for the test are given in brackets next to the patient’s results. According to the guidelines of the Polish Diabetes Association, the result of such a test after 2 h should optimally be below 140. A result of over 200 indicates diabetes. The recorded result was almost twice as high, indicating diabetes and an urgent need for pharmacological intervention.

	0 min.	30 min.	60 min.	120 min.	180 min.	240 min.
Glucose (mg/dL)	124 (N: 70–99)	217	260	**373**	299	189
Insulin (μIU/mL)	2 (N: <29)	3.52	2	5.95	11.2	75.1

**Table 4:** Biochemical blood test for insulinoma.

Blood Test	Result
Glucose	33 mg/dL
Insulin	27.1 μIU/mL
C-peptide	6.8 ng/mL

**Table 5:** Results of the test with short-acting octreotide—100 µg s.c.

	0 min.	60 min.	120 min.	180 min.	240 min
Glucose mg/dL	160	137	158	143	133
Insulin μIU/mL	<2	<2	<2	<2	<2

**Table 6:** The results of the test blood markers associated with NETs from June 2023. The conducted blood assay consists of chromogranin A, neuron-specific enolase (NSE), and 5-hydroxyindoleacetic acid (5HIAA).

	Result	Normal Range
Chromogranin A	337.3 µg/dL	<100 µg/dL
NSE	75.85 ng/dL	<18.3 ng/dL
5HIAA	7.15 mg/24 h	2–9 mg/24 h

**Table 7:** G3 neuroendocrine tumor in breasts—results from August 2023, Lower Silesian Pulmonology and Hematology Oncology Center.

	Ki67	Synaptophysin	Chromogranin A
Left side	30%	+	+
Right side	50%	+	+

**Table 8:** Presentation of the case description in the form of a table.

Date	Event
2009	First symptoms: abdominal pain; pancreatic tumor confirmed in USG examination
July 2009	Distal pancreatectomy with splenectomy; histopathological exam: solid pseudopapillary tumor of the pancreas
September 2019	Segmentectomy (S5-S4b) with cholecystectomy with the histopathological recognition of neuroendocrine tumor (NET) metastasis to the liver characterized as NET G2, Ki67—5%
January 2022	Short episode of loss of consciousness with convulsion due to hypoglycemia; repeated states of hypoglycemia—hospitalization at the Neurology Department in Wroclaw
April 2022	Diagnostic at the Department of Endocrinology, Diabetes and Isotope Therapy in Wrocław—72 h fasting test excluding insulinoma; diagnosis of type 3 diabetes; metastatic process found in the liver in PET-FDG
May 2022	Persistent hypoglycemia with elevated insulin and C-peptide levels → diagnosis of insulinoma; start of treatment using somatostatin and diazoxide; metastatic tumor in segment I of the liver confirmed in MRI
June 2022	EUS with hepatic biopsy → NET with higher malignancy, G2/G3, Ki67—up to 20%.
August 2022	Median upper relaparotomy with alcoholization of the liver tumor
November 2022	Follow-up CT showing new metastases in S2, S3, and S6 of the liver
January 2023	Follow-up PET showing foci in the liver, head of the pancreas, bones, and cervical lymph nodes
May 2023	Beginning of Lutathera treatment CT scan: metastases in the liver, bone sclerosis, enlarged lymph nodes
June 2023	Administration of continuous glucose monitoring with FSM Breast ultrasound and mammography: lesion found in both of the mammary glands (right breast—BIRADS4; left breast—BIRADS2)
July and August 2023	Biopsy of the mammary glands: metastases with high proliferation markers (triple negative malignant neoplasm) NET G3, Ki67—30% for left breast and 50% for right breast Administration of a second dose of Lutathera
October 2023	Significant progression of metastases in the liver and bones (spine, ribs, pelvis), and periaortic and pelvic lymphadenopathy The patient was referred to palliative hospice care, resigned from further PRPRT treatment
November 2023	Chemotherapy with temozolomide—I cycle
27 December 2023	Patient died

**Table 9:** A compilation of cases of patients with neuroendocrine tumors of the insulinoma type described in recent years. The patients are described according to their age, sex, and place of origin. The main focus is on the description of symptoms, procedure, and follow-up. The table was prepared based on the work of [22,32,35,39,41,42,43,44,45,46,47,48,49,50].

Similar Cases					
	Patient Data	Symptoms	Type of Treatment	Effect of Treatment	Citation
1	A 16-year-old male, South America	hunger sweating headache lack of concentration irritation tremor muscle weakness visual disturbances	Surgical treatment—enucleation of the tumor. No metastases were detected.	The patient feels a significant improvement in his condition.The symptoms have completely disappeared.	[45]
2	A 47-year-old female, Asia	somnambulism half-hour seizure tachycardia and dizziness fatigue	Surgical treatment—laparoscopic partial distal pancreatectomy. No metastases were detected.	Three months after surgery, the patient reported feeling well, and follow-up tests (glucose, insulin, C-peptide) showed normal results.	[46]
3	A 41-year-old female, Europe	recurrent hypoglycemic episodes diuretic-resistant edema	Surgical treatment—distal pancreatectomy and splenectomy. Pharmacological treatment: - Lanreotide autogel - Diazoxide - Dexamethasone - Everolimus - Intravenous glucose and subcutaneous glucagon Selective internal radiation therapy (Y-90) for liver metastases. Hepatic and lymph node metastases have been reported. Tumor infiltration of the perihilar tissues was found.	The patient experienced respiratory distress likely due to infection or drug-associated pneumonitis. She developed acute respiratory distress syndrome. Unfortunately, she died three months after the initial diagnosis due to ARDS.	[47]
4	A 26-year-old female, Africa	recurrent episodes of loss of consciousness confusion seizures hallucinations polyphagia significant weight gain	Surgical treatment—enucleation of the tumor. No metastases were detected.	Following the surgery, the patient had a full recovery. The patient no longer experienced symptoms such as fatigue, increased appetite, seizures, or loss of consciousness.	[48]
5	A 43-year-old female, Asia	sudden onset of lightheadedness and shakiness increased appetite over the past year recent weight gain of 15 kg	Surgical treatment—enucleation of the tumor. No metastases were detected.	After the surgical treatment, her glucose levels rose to the diabetic range, suggesting that the hypoglycemic symptoms were effectively managed.	[49]
6	A 38-year-old female, Europe	multiple episodes of malaise fatigue fainting	Surgical treatment—laparoscopic partial distal pancreatectomy. Pharmacological treatment: - Diazoxide No metastases were detected.	After surgery, the patient’s insulin, proinsulin, C-peptide, and glucose levels returned to normal. After 16 months, the patient had lost 4.2 kg and did not report any specific complaints.	[50]
7	A 53-year-old male, Europe	weakness and fatigue shakiness and trembling hunger	Surgical treatment—removal of the tumor. No metastases were detected.	After the surgery, all symptoms subsided, and the patient did not require any additional treatment.	[41]
8	A 14-year-old female, Asia	abnormal behavior during sleep, including the inability to wake up, eyes rolling upwards, and limb twitching inability to wake up for extended periods, such as 12 h of sleep from night to afternoon excessive daytime sleepiness and abnormal behavior such as confusion and poor memory	Surgical treatment—laparoscopic partial distal pancreatectomy. No metastases were detected.	Symptoms improved significantly, including the disappearance of daytime sleepiness and abnormal behavior during sleep. The patient’s blood glucose levels normalized.	[42]
9	A 64-year-old female, Europe	neuroglycopenic symptoms, which resolved with meal	Pharmacological treatment: - Somatostatin analog—octreotide - Diazoxide and underwent trans-arterial chemoembolization (TACE) - Lanreotide - Everolimus - Sunitinib - Capecitabine and temozolomide (CAPTEM) - Pasireotide LAR The presence of liver metastases was found.	Patient’s glycemic control improved significantly, and hypoglycemic episodes became much less frequent and severe. Blood glucose levels were completely normalized for over 18 months, resulting in a significant improvement in her quality of life.	[43]
10	A 55-year-old female, Europe	episodes of altered consciousness, lasting approximately 30 min general malaise weakness difficulty or inability to speak sweating partial amnesia	Surgical treatment—removal of the tumor. Pharmacological treatment: - Diazoxide No metastases were detected.	Postoperatively, the patient’s glucose level and insulin regulation normalized, leading to an overall improvement in her health.	[44]
11	A 65-year-old male, South America	loss of consciousness after meals spontaneous hypoglycemia recovery after consuming glucose	Surgical treatment—Subtotal pancreatectomy (90%) with splenectomy. No metastases were detected.	Improvement in glucose control after surgery. Initial postoperative hyperglycemia managed with insulin	[22]
12	A 54-year-old male, Asia	sweating weakness mood changes	Pharmacological treatment: - 177Lu-DOTATATE therapy (radiolabeled somatostatin receptor therapy). Metastatic lesions in the right lung and liver were detected.	After treatment, the size and activity of the lesions decreased. Hypoglycemic episodes, which previously occurred daily, decreased to 1 episode per year during a 1-year follow-up.	[32]
13	A 72-year-old female, Europe	excessive sweating weakness trembling, anxiety symptoms were triggered by agitation and excessive sweating during hypoglycemic episodes	EUS-guided ethanol ablation (two sessions). No metastases were detected.	After the first session, partial improvement was observed: hypoglycemic episodes occurred less frequently, and the symptoms were milder. After the second session, the treatment appeared to be fully effective, with no hypoglycemic episodes for five months. Adverse effects: mild abdominal pain on the day of the procedure and a transient six-fold elevation of lipase activity, which normalized within 72 h.	[35]
14	A 60-year-old male, North America	sweating tremors mental deterioration pathological bone fractures as a complication of metastasis	Pharmacological treatment: - Diazoxide - Chemotherapy with the use of streptozotocin, 5-FU, etoposide, and cis-platinum Irradiation of metastatic bone lesions was performed. Metastatic lesion was demonstrated in the liver and bones.	Despite the treatment administered, hypoglycemic symptoms progressed. Sepsis appeared. The patient died after 11 months of diagnosis.	[39]

## Data Availability

The original contributions presented in this study are included in the article. Further inquiries can be directed to the corresponding author.
